# Dermatological Brevities

**Published:** 1907-06-22

**Authors:** 


### DERMATOLOGICAL BREVITIES.

### Vaso-Motor Disorders.

Common urticaria is, perhaps, the affection which illustrates best the action of the vaso-motor nerves upon the cutaneous blood-vessels. The stimulus may arise from within, as, for instance, the irritation caused by the absorption of some toxin from the alimentary canal; or it may come from without, as when the surface is sharply stroked in order to demonstrate the presence of dermatographia. Whether the bright red patch or the livid streak subsists, the paralytic effect upon the vessels is the same. Excessive flushing after meals, constituting the mildest degree of rosacea, is another manifestation of the same morbid process. Ichthyol, given in pill form, in doses of five minims, twice or three times a day, is one of the most useful drugs for restoring vaso-motor tone. At the same time, it is important to ensure the free and regular action of the bowels.

### Feigned Eruptions.

Whenever there exists an extraordinary-looking eruption, more or less inflammatory in character, situated within easy access of the hands, corresponding to no known skin disease, and occurring in an otherwise healthy but neurotic young woman, the presumptive evidence is very strong that it has been artificially produced by the sufferer herself. It is difficult, and in many cases impossible, to bring the charge home to the person concerned, but a fairly certain diagnosis may be made if the eruption rapidly improves under the application of a simple soothing ointment, the dressing of which is fixed in place under plaster of Paris and only removed by the doctor in the presence of the nurse. Many and varied are the agents which have been employed in the production of a *Dermatatis artefacta*, from maceration and friction of the integument with the patient's own saliva to the actual application of a live coal or of crude vitriol.

### Formalin for Sweating.

Excessive sweating of the palms, feet, and axillæ is not infrequently met with in anæmic individuals, and is a most troublesome symptom. Bathing the affected parts with a sponge wrung out of very hot water and applying immediately afterwards a 5-per-cent. lotion of formalin may be tried. After the latter, a dusting powder of chalk, alum, and starch, in the proportion of one, two, and three, may be shaken on. Constitutional treatment with iron and strychnine is nearly always indicated in addition. One gratifying result of the formalin treatment is that the drug is such a good deodoriser.

### The Diagnosis of Scabies.

The only strictly scientific diagnosis of the itch is the demonstration of the *Acarus scabiei* under the microscope. In cleanly patients it may be difficult, or even impossible, to see a single characteristic "burrow." The best guide in such cases is the distribution of the lesions these may be found; not only in the common sites, such as the interdigital clefts and fronts of the wrists, along the margin of the anterior axillary fold, and in sub-mammary crease and the umbilicus. If sulphur be objected to, a little balsam of Peru or epicarin may be used instead.

